# Supplementary material for: Active household contact screening for tuberculosis and provision of isoniazid preventive therapy to under-five children in Afghanistan
Source: PLoS One. 2020 Oct 9;15(10):e0240031. doi: 10.1371/journal.pone.0240031 (PMC7546473; doi:10.1371/journal.pone.0240031)
Supplement: Attachment — Submitted filename: Contact Screening Afghanistan response to reviewers.docx [file pone.0240031.s002.docx]

**Response to Reviewers’ Comments on**

**Active household contact screening for tuberculosis and provision of isoniazid preventive therapy to under-five children in Afghanistan**

**Reviewer #1:** **This is an interesting study of secondary data on contact investigation, a high priority according to the UN high-level meeting held in NYC in 2018. Two previous studies have reported the prevalence of active TB among contacts in Afghanistan. The current manuscript adds a lot to these reports, by discussing LTBI treatment, uptake and completion, and by describing the substantial increase in case finding after the implementation of a new policy in the country. These findings are interesting. The manuscript, however, needs some improvements before it can be suitable for publication.**

**Abstract**
**This abstract should be rewritten. The objectives should clearly state that a new policy was recommended by the NTP in 2011 for contact investigation and this observational study reports the findings since these recommendations.**

*We have rewritten the abstract to clarify that this study came after the new NTP policy and reports findings subsequent to those recommendations*.

**The Methods section currently describes mainly active TB indicators (NNS and NNT) but the conclusions focus on IPT in children. The authors need to choose what their main message is and write a coherent abstract. If the number of children starting and completing LTBI treatment is their main focus, they need to define what the national policies for contact investigation and treatment for LTBI were in 2011-2018 in the Methods section, so that the reader can understand why so many children under 5 were prescribed IPT.**

*We presented the yield of contact screening and LTBI treatment among under-fives in the article to show that, without strong contact screening, LTBI for those young children will be high. We wanted to demonstrate that contact screening is an entry point for IPT as well as for increased case notification. The title and the objectives reflect this interrelationship.*

**In the conclusion, what do the authors call a “high” performance? Do they mean good? The findings should be interpreted in the same way they are reported in the Results section. Incidence rate is not shown in results, so the reader can not follow the conclusion. Please adjust.**

*We have included the missing figures*.

**The conclusion should also describe briefly how the authors explain the high rates of IPT prescription and completion when compared to other countries.**

*We have rewritten the conclusion to explain the high rates of IPT prescription and completion*.

**The sentence ending the abstract is misleading: Afghanistan is not a HBC, and the sentence refers to “other” HBC. Afghanistan is a high TB incidence RATE country, which is different from HBC. Please adjust.**

*Although Afghanistan is not in the WHO HBC category, the incidence of 189/100,000 is higher than in some of the HBCs. We have corrected the comparison with HBCs.*

**The short title should be adjusted. The study is not about children only.**

*We have revised the short title as follows: Household TB contact screening, and preventive therapy for children, in Afghanistan*

**Main text:**

**Introduction**
**This report is of interest for many other countries. Accordingly, the introduction should highlight why the investigation of contacts is important (anywhere), and just a short paragraph to explain why it is important in Afghanistan. The detailed description of the TB situation in Afghanistan should be reported in a “setting” subheading of the Methods section.**

*Thank you for this recommendation. We have updated the introduction and methods sections accordingly.*

**L. 32-33: Incidence and incidence rates are different concepts, please rephrase.**

*We have reworded this sentence.*

**Methods
This is an observational study of a new policy recommended by the NTP. The new policy and the routine investigation of contacts should be explained but not be named “intervention” because this is misleading. All that is currently reported as the “intervention” should be reported as setting. This section should also make clear what were the previous recommendations and what is new in the current recommendation**.

*We have corrected the text to issue these issues.*

**Are the reported definitions the ones adopted by the NTP? Are health care workers and community agents instructed to identify contacts as per this definition?**

*We have added details to the section on definitions to address these questions.*

**Data analysis: time trend would be a better analysis (quarterly, since these data is available by quarter) than proportions. The increase (or any change) in the population (denominator) should be taken into consideration in analyses/comments of incidence rates.**

*We have included a trend analysis for all forms and bacteriologically confirmed TB.*

**Ethics: how was data stored? What procedures were made to protect the data?**

*We revised lines 139-42 as follows: “We obtained approval from the Afghanistan NTP to use the data collected and aggregated in the MOPH Management Information System database, and no additional ethical approval was required because we used de-identified data collected from routine programmatic reports of the NTP.”*

**Results**
“**We included” is not a good term for an analysis of secondary data.**

*We revised this wording.*

**The p-values are unnecessary, since the CI are presented (and any difference with such a huge sample would be “statistically significant**”).

*Thank you; we have removed the p values.*

**Discussion
The results should not be repeated in the discussion.
Instead of simply comparing the yield of active TB among screened** **contacts, it would be interesting to discuss what might explain these differences.**

*We explained in our discussion what should be described for the observational study. We do not know what additional comparisons to offer, based on the nature of the study.*

**What does this manuscript add to the two previous reports on contact investigation in the country? This should be highlighted in the discussion. How do the current findings compare to findings before the new policy? In other words, what is the impact of the new policy? Were there any other changes in the country or health system in this period that could explain the increase in the proportion of TB cases diagnosed?**

*There was no data on contact screening and IPT organized data before 2011, so it is difficult to compare. But we compared the results of the study done in Afghanistan with ours. We also updated the policy implications for the future of the country.*

**Comments on cost-effectiveness of the NNS and NNT would be interesting, even if speculative since this kind of analysis was not the scope of the study.**

*It is difficult to comment on cost-effectiveness at this point and with the data we have. We agree that it would be an interesting focus of future studies.*

**Above all, the conclusions cannot be withdrawn from the findings of the study.**

*We revised some aspects of the conclusion that were speculative.*

**Minor comments:**
**1. The text needs some language review.**

*The paper has been re-edited by a professional editor.*

**2. References need to be updated.**

*We have checked and updated all references.*

**Reviewer #2: Summary of Paper:**

**The manuscript summarizes 8 years of national TB program data in Afghanistan. During this time period 142,797 active TB cases were reported and 586,292 household contacts of index cases were screened for TB. The number of co-prevalent cases among HHC was found to be high. TPT initiation and completion rates for children under five were high. In many cases data is reported separately for men and women. The number needed to screen (NNS) and number needed to treat (NNT) are also reported.

General comments:
Overall this was an informative and well written paper. Data is presented for many useful TB indicators. The performance of the program is remarkable. Some additional explanation as to how the program has been able to perform so well would be useful so that others can learn from the experience in Afghanistan.**

*We expanded on our experience in the methods and discussions sections.*

**Major comments:
1. On page 4 the authors state that they use “routine contact screen performance” data from the Afghanistan NTP from 2011-2018. It would be useful to know more about how this data is collected and how good the data is felt to be? (has the same registry been in place since 2011 to collect this data? Are they electronic registries? Do they combine data from multiple registries? How reliable are the data collected in screening registries? How complete are they?)**

*There was no electronic register, only paper-based registration for contact screening and IPT. Because the project was supported by USAID, there was a robust data quality assurance mechanism, which we have described a little more in the revised Methods section. The register was developed in 2011.*

**2. Related to the point above, within these registries how is treatment completion measured?**

*A definition is included in the Methods: “**IPT completion is defined as the completion of more than 95% of the prescribed INH dose, ascertained by TB focal persons by counting the pills in a blister pack, and DOT by community health workers when they observe the treatment of the index case.”*

**3. How large is the private sector in Afghanistan? If the private sector is involved in TB care, how is this captured in the data reported? Is the private sector involved in routine program reporting? The role of the private sector and the generalizability of the findings reported in the manuscript data should be included in the discussion.**

*The private sector is small. Some private providers are engaged in TB diagnosis and treatment in the capital, but they do not usually conduct contact screening, so the data is for the public sector only.*

**4. The authors show that there have been major improvement in the TB outcomes reported during this period. It would be interesting to know what mechanisms may have contributed to the major improvements seen in the country in the last few years. For example has program funding remained stable over these years? How/why has it changed? Have strengthening projects been in place? Readers will be interested to know what investments are required in order to bring a program to this level (both in terms of both $ and program strengthening through Challenge TB etc…)**

*There were substantial investments, mostly from USAID and the Global Fund. Because of this support, capacity building—in terms of training, mentorship, diagnostics, and drug supplies—improved enormously. The relative improvement in the security of the country was another factor in the good performance. These are some reasons for the improvements, which we have elaborated on in the section on the setting.*

**5. Throughout the manuscript, the authors stratify most findings by sex, and use statistical testing to look at sex differences. It would be helpful to provide a justification in background to support these analyses. Was there an initial hypothesis that there would be gender differences which led to these analysis? I’m not sure what the statistical testing adds without a clear discussion of the rationale and implications. In the discussion there is currently just a comparison to what others have found.**

*Because there is a difference in access to health care for men and women in Afghanistan, we stratified the analysis by sex and have briefly explained this in the data analysis.*

**6. In the paper the authors use census data to estimate 6 household members. Do the authors know how many HHC were usually identified when contact investigators were performed? Is there any data available to validate the census assumption?**

*We used the national average household estimate because the register did not capture household members of index TB cases. It captured only those screened and is, therefore, a limitation of the article, as noted in the section on limitations. We are recommending inclusion of the household members of index TB cases for the future.*

**7. In the discussion the authors state that they attribute the high rate of completion to various things (counseling of parents on the importance of IPT etc..). It would be nice to have more detail about what is involved programmatically in achieving this level of passive case detection, screening, testing and treatment initiation, so that others can learn from the successes of this program. For example, what is involved in good follow up? What lengths do HCW go to in order to help people initiate and complete IPT. If the authors can elaborate on any of these points it would strengthen the paper.**

*In the discussion, we have provided more details to explain that the mandatory contact screening of all index cases following NTP guidance and capacity building are the reasons for the high achievement. The advantage with TB is that the treatment is DOT, so the index case stays within the health care system for six months until they complete treatment. In all the daily contacts in this period, patients are asked if all their contacts have been screened.*

**8. The authors’ final statement that IPT “should probably be extended to all eligible contacts of all ages” seems like a bit of an afterthought. For a program that is seemingly performing so well, one could argue this would be an obvious next step. Can the authors comment on if there are plans for this, or if there are obstacles? It seems that this notion deserves more attention, especially in light on the UNHLM targets to expand TPT to 20 million HHC of all ages by 2022.**

Although expansion of TPT is now a global recommendation, its success will depend on factors such as the availability of IPT. We will support the NTP to plan to progress toward the new direction in the coming years.

**Minor points:**
**9. line 110- it should be made clear that by “contact” they are referring to household contacts (based on the definition provided). Throughout would suggest referring to household contact for clarity.**

*Thank you. We have clarified this.*

**10. line 114 there is a typo?- should be NNS (not NNT)**

**11. Line 235- typo? It states “interrupted” supply of INH- presumably it should say “uninterrupted”.**

*We have corrected these typos.*

**12. Page 6 the first line on page 7 (line 132) is redundant as it has already been stated in the first line of the results**
*We have deleted the repetition.*

**13. Page 13, line 251- we recommend following contacts for 2-3 years- to monitor for TB? Pls clarify for what reasons**

*We clarified that the reason for this recommendation is because if patients develop TB it happens within 3 years.*
